# Investigating causal associations between serum metabolites and cataract by Mendelian randomization

**DOI:** 10.1097/MD.0000000000046662

**Published:** 2026-01-02

**Authors:** Xiaoying Yang, Jianzhong Mei

**Affiliations:** a Department of Ophthalmology, Taixing People’s Hospital, Taixing, China.

**Keywords:** carnitine metabolism, cataract, citrate, Mendelian randomization study, serum metabolite

## Abstract

Early studies have demonstrated that systemic metabolic changes can significantly contribute to the development and progression of cataract, however, the causal effects of specific human serum metabolites on cataract have not been fully elucidated. The causal effects of 7 serum metabolites, including proline, serine, 3-dehydrocarnitine, N-[3-(2-oxopyrrolidin-1-yl)propyl]acetamide, octanoylcarnitine, decanoylcarnitine, and citrate, on cataract were analyzed using a 2-sample multivariable Mendelian randomization (MR). Using data from multiple genome-wide association study datasets, which fall under the broad category of the studied serum metabolites, we identified instrumental variables following strict criteria. We then analyzed a FinnGen genome-wide association study dataset, which included 39,519 European cataract cases and 452,358 European controls, employing the inverse variance weighted method, and rigorous tests for horizontal pleiotropy and heterogeneity as well as sensitivity analysis, to ensure robustness of the results. The MR analysis uncovered that 3-dehydrocarnitine and citrate have significant positive causal associations with cataract, while proline, serine, N-[3-(2-oxopyrrolidin-1-yl)propyl]acetamide, octanoylcarnitine, and decanoylcarnitine, have significant negative causal associations with cataract (*P* < .05). Notably, no significant pleiotropy or heterogeneity was detected (*P* > .05) in the MR analysis, and sensitivity analysis confirmed the robustness of the results. This research uncovered significant causal associations between several serum metabolites and cataract, indicating altered metabolic pathways involved in cataract pathogenesis. These findings further suggest the studied serum metabolites may serve as biomarkers for early disease diagnosis and highlight promising targets for developing effective management and treatment strategies in cataract.

## 1. Introduction

Cataract is a medical condition in which the lens of the eye becomes progressively opaque, resulting in impaired vision. It represents one of the leading causes to blindness, where over 50% of all cases of blindness are attributed to cataract.^[1]^ In the US alone, it is estimated that more than 25 million people suffer from cataract, and this number is projected to 38.5 million by the year of 2032, and to 45.6 million by the year 2050.^[2]^ There are no existing therapeutics for cataract, and surgery is the only treatment during which the damaged lens is replaced by an artificial intraocular lens, thanks to the most cutting-edge surgical techniques and intraocular lens technology. Thus, a better understanding of the distinct genetic and pathophysiological factors influencing cataract is vital for improving diagnosis and therapeutic development, given its significant global health burden.

The pathogenesis of cataract is multifactorial. However, blood serum metabolites have particularly gained increasing attentions as they have been implicated in the development and progression of cataract. For instance, Yin et al reported that hyperlipidemia is the main factor for increased lens density in patients with age-related nuclear cataract, where serum total cholesterol, triglyceride, low-density lipoprotein cholesterol levels correlate with lens density.^[3]^ In addition, Glaesser et al reported that an increased molar ratio of free fatty acids to albumin in blood can cause mitochondria-mediated apoptotic death of lens epithelial cells, lens opacification, and cataract.^[4]^ Oxidative stress is another critical risk factor of cataract, as Chang et al reported that the serum levels of oxidative stress-associated advanced oxidation protein products, protein carbonyl, and 8-hydroxydeoxyguanosine are significantly higher in patients with cataract compared to healthy individuals.^[5]^ All these aforementioned and other pioneering studies highlight the relevance of serum metabolites to cataract, and there has been a strong impetus on studying the causal effect of serum metabolites involved in various metabolic pathways on cataract.

While animals differ from humans in genomes, physiology and anatomy, and patients are often demographically and clinically heterogeneous, it is difficult to uncover the causal effect of serum metabolites on cataract in animal research (failure for human translation) or traditional observational studies (significant confounding and reverse causality). Meanwhile, Mendelian randomization (MR) analysis is an epidemiological method that leverages genetic variants from genome-wide association studies (GWAS), typically single-nucleotide polymorphisms (SNPs), as instrumental variables (IVs) to simulate randomization and to establish causality between exposures and outcomes.^[6]^ Importantly, MR analysis can avoid reverse causality as genotype formation precedes disease onset and is unaffected by disease progression, medication or other environmental factors which can otherwise obscure the true causal associations, thus providing an ideal solution for the causality study.^[7,8]^

This research aimed to employ a 2-sample MR analysis to investigate causal associations between certain serum metabolites and cataract that are poorly understood, using the latest publicly available GWAS datasets. Seven serum metabolites, including proline, serine, 3-dehydrocarnitine, N-[3-(2-oxopyrrolidin-1-yl)propyl]acetamide, octanoylcarnitine, decanoylcarnitine, and citrate, which are critically involved in redox balance, mitochondrial function, and amino acid and fatty acid metabolisms, and therefore may contribute to retinal health and diseases,^[9–15]^ were selected for analysis. Findings from this research not only help to recognize the metabolic mechanisms underlying cataract, but also provide reliable evidence for establishing feasible strategies for early diagnosis and prevention of cataract in clinical practice.

## 2. Methods

### 2.1. Study design

This research employed a 2-sample MR analysis to explore the causal associations between certain serum metabolites (the exposure) and cataract (the outcome). The workflow followed the STROBE-MR (strengthening the reporting of observational studies in epidemiology using Mendelian randomization) guidelines for reporting MR research clearly and transparently.^[16]^ To enhance the validity of the findings and reduce the impact of extraneous factors, the MR analysis adhered to the following core assumptions^[7]^:

1. The SNPs selected as IVs exhibit strong associations with the studied serum metabolites;
2. The IVs are independent of confounders that could introduce bias;
3. The IVs affect the outcome solely through the exposure, ensuring no direct effects on cataract beyond their association with serum metabolites.

**Figure 1.**
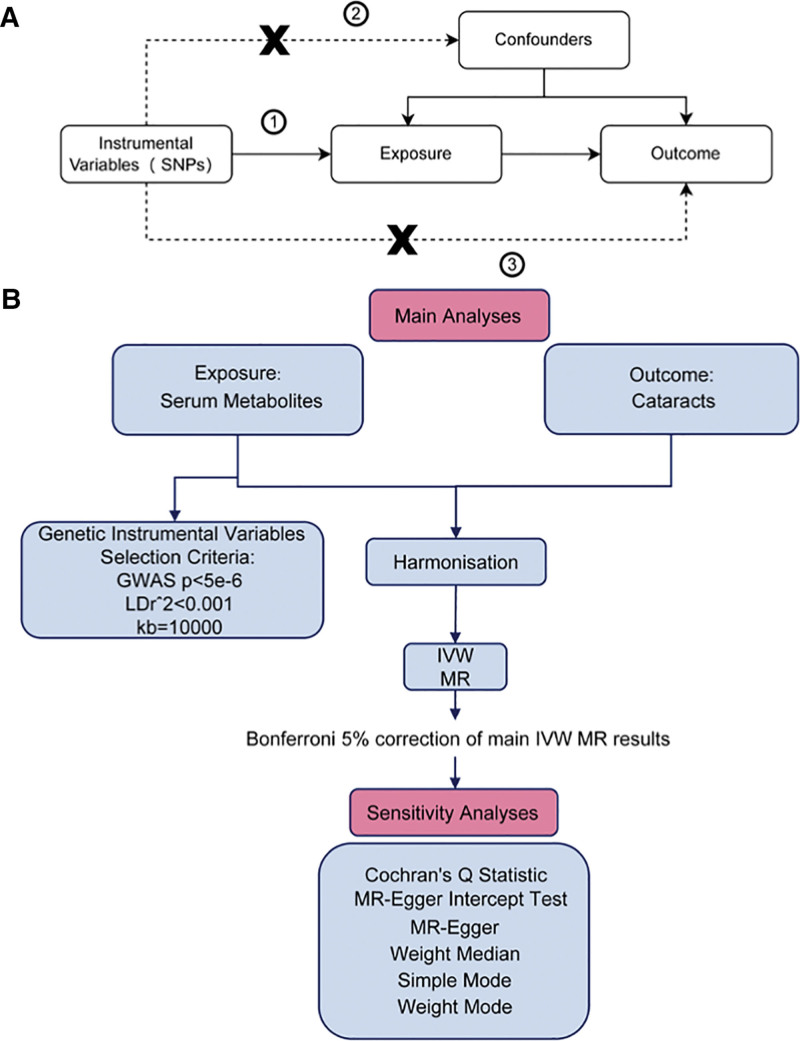
Flowchart of the MR analysis. IVW = inverse variance weighted, GWAS = genome-wide association study, LD = linkage disequilibrium, MR = Mendelian randomization.

Figure 1 displays a flowchart of the MR analysis.

### 2.2. Data sources and ethics statement

This research analyzed publicly available datasets. Specifically, we focused on 7 serum metabolites, including proline, serine, 3-dehydrocarnitine, N-[3-(2-oxopyrrolidin-1-yl)propyl]acetamide, octanoylcarnitine, decanoylcarnitine, and citrate, and the genetic data were obtained from GWAS datasets ID: met-a-355, met-a-464, met-a-500, met-a-505, met-a-618, met-a-615, met-c-849. Year of serum metabolite datasets, sample size, and number of SNPs were summarized in Table S1, Supplemental Digital Content, https://links.lww.com/MD/Q963. The studied serum metabolites were selected based on the current literature, where these serum metabolites potentially play critical roles in in retinal health and diseases.^[9–15]^ On the other hand, the cataract genetic data were obtained from GWAS dataset ID: GCST90018814, which includes 39,519 European cataract cases and 452,358 European controls.^[17]^ Since these datasets are publicly available and have already received ethical approval from the respective institutions at the time of study implementation and data collection, no further ethical approval was necessary for this reanalysis.

### 2.3. Selection of instrumental variables

SNPs are the most common genetic IVs in the human genome. SNPs that were significantly associated with exposures (*P* < 5 × 10^‐6^) were selected for further analysis. To ensure the robustness of causal inferences regarding the association between the studied serum metabolites and cataract, we implemented several quality control procedures to select optimal IVs. Initially, linkage disequilibrium clumping was applied to these SNPs using a stringent threshold of *R*^2^ = 0.001 within a 10,000 kb window. Additionally, to assess the strength of the selected IVs and minimize weak instrument bias, the *F*-statistic was calculated and the SNPs with an *F*-statistic of below 10 were considered weak and subsequently excluded.^[18]^

### 2.4. Statistical analysis

The causal effects of the studied serum metabolites on cataract were primarily assessed using the inverse variance weighted (IVW) method, which assumes no average pleiotropy and nonsignificant heterogeneity.^[19]^ Horizontal pleiotropy was evaluated using the MR-Egger intercept test, where *P* > .05 indicated no significant pleiotropy. Heterogeneity across SNPs was assessed using Cochran *Q* test, and if significant heterogeneity was detected (*P* < .05), a multiplicative random-effects IVW model was applied to provide a more conservative and reliable estimate; otherwise, a fixed-effects IVW model was applied.^[20]^ To enhance the robustness of the causal inference and minimize the impact of weak instrument bias and reverse causality, we applied complementary MR methods and conducted additional sensitivity analyses. We carried out: weighted median analysis, which provides a consistent estimate even if up to 50% of the instruments are invalid; MR-Egger regression, assessing the pleiotropy and providing bias-corrected causal estimates; and simple mode and weighted mode analyses, which offers additional robustness checks.^[21]^ We finally performed leave-one-out analysis, which assesses the robustness of the causal estimate by sequentially excluding each SNP from the instrument set. This approach is crucial in determining whether the observed association is driven by any single influential SNP and evaluates the overall stability of the MR results.^[22]^

All data calculations and statistical analyses were performed using R software (https://www.r-project.org/) (version 4.3.0). Effect estimates were reported as β values with 95% confidence intervals (CIs) and converted to odds ratios (ORs) through exponential transformation, providing an intuitive interpretation of the results. All statistical tests were 2-tailed, and *P* < .05 was considered as the significant level.

## 3. Results

### 3.1. Instrumental variables in the studied serum metabolites

A range of 3 to 6 SNPs were pinpointed as potential proxies for individual serum metabolites under investigation through stringent criteria, including genome-wide significance threshold (*P* < 5 × 10^‐6^), harmonization, linkage disequilibrium analysis, and *F*-statistics. All remaining SNPs had *F*-statistic values of >10, indicating that the SNPs were strong-effect IVs, and the possible bias caused by weak IVs was limited. The final compilation of selected SNPs and *F*-statistics were also summarized in Table S1, Supplemental Digital Content, https://links.lww.com/MD/Q963.

### 3.2. Causal effects of the studied serum metabolites on cataract

**Figure 2.**
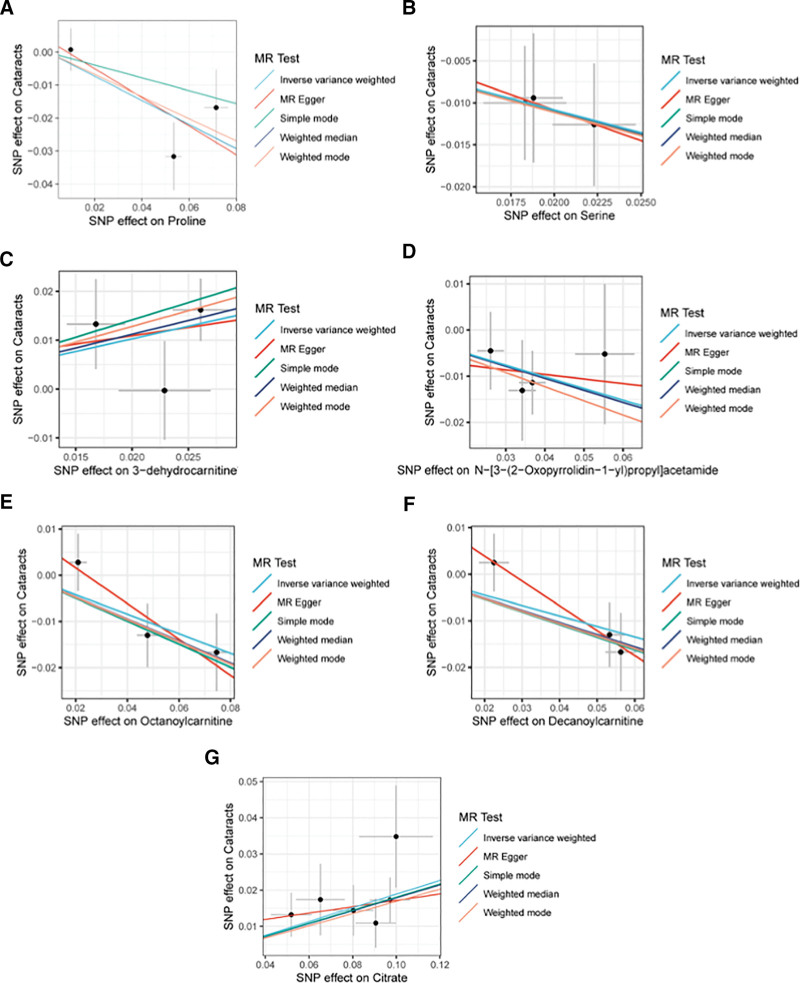
Scatter plots of MR analysis results on causal associations between the studied serum metabolites and cataract. Plots showing SNP effects on (A) proline, (B) serine, (C) 3-dehydrocarnitine, (D) N-[3-(2-oxopyrrolidin-1-yl)propyl]acetamide, (E) octanoylcarnitine, (F) decanoylcarnitine, (G) citrate, and cataract. MR = Mendelian randomization, SNP = single-nucleotide polymorphism.

**Figure 3.**
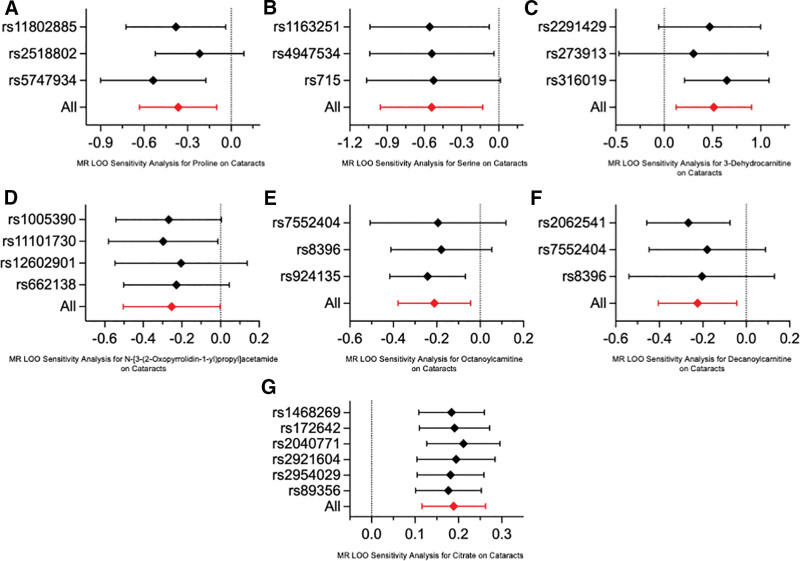
Stability of causal associations between the studied serum metabolites and cataract assessed by MR LOO sensitivity analysis. The *x*-axis shows the MR LOO sensitivity analysis of (A) proline, (B) serine, (C) 3-dehydrocarnitine, (D) N-[3-(2-oxopyrrolidin-1-yl)propyl]acetamide, (E) octanoylcarnitine, (F) decanoylcarnitine, and (G) citrate on cataract. The *y*-axis shows the analysis of the effect of removing individual SNPs. LOO = leave-one-out, MR = Mendelian randomization, SNP = single-nucleotide polymorphism.

MR analysis by IVW method revealed that a genetically predicted higher relative abundance of proline (OR = 0.69, 95% CI = 0.53–0.91, *P* = .0071), serine (OR = 0.58, CI = 0.38–0.88, *P* = .0101), N-[3-(2-oxopyrrolidin-1-yl)propyl]acetamide (OR = 0.78, CI = 0.60–1.00, *P* = .0476), decanoylcarnitine (OR = 0.81, CI = 0.69–0.96, *P* = .0130) or octanoylcarnitine (OR = 0.80, CI = 0.67–0.96, *P* = .0149) is associated with a significantly lower risk of cataract, while a genetically predicted higher relative abundance of 3-dehydrocarnitine (OR = 1.67, CI = 1.13–2.47, *P* = .0101) or citrate (OR = 1.21, CI = 1.12–1.30, *P* < .0001) is associated with a significantly higher risk of cataract (Fig. 2). Moreover, heterogeneity across the SNPs was analyzed by Cochran *Q* test, where no significant heterogeneity was found (*P* > .05). The MR-Egger intercept analysis further indicated the absence of horizontal pleiotropy (*P* > .05). These key analysis results were summarized in Table 1. Furthermore, leave-one-out sensitivity analysis confirmed that no single SNP disproportionately influenced the findings (Fig. 3). Additional MR analysis results employing MR-Egger, weighted median, simple mode and weighted mode methods were summarized in Table S2, Supplemental Digital Content, https://links.lww.com/MD/Q963. Altogether, these analyses ensured the robustness of our findings on the causal effect of the studied serum metabolites on cataract.

**Table 1:** MR analysis of causal effect of the studied serum metabolites on cataract.

	MR analysis								Heterogeneity and pleiotropy analysis				
Exposure	Method	No. SNPs	Beta	SE	*P*-value	OR	95% CI	Method		*Q*	*P*-value	Egger-intercept	*P*-value
Proline	IVW	3	0.365	0.136	.0071	0.69	0.53–0.91	IVW and MR-Egger		2.5	.286	3.70 × 10^‐3^	.802
Serine	IVW	3	0.542	0.211	.0101	0.58	0.38–0.88	IVW and MR-Egger		0.0156	.992	3.78 × 10^‐3^	.948
3-Dehydrocarnitine	IVW	3	0.514	0.200	.0101	1.67	1.13–2.47	IVW and MR-Egger		1.87	.392	4.09 × 10^‐3^	.932
N-[3-(2-Oxopyrrolidin-1-yl)propyl]acetamide	IVW	4	0.253	0.128	.0476	0.78	0.60–1.00	IVW and MR-Egger		0.655	.884	5.62 × 10^‐3^	.812
Octanoylcarnitine	IVW	3	0.211	0.085	.0130	0.81	0.69–0.96	IVW and MR-Egger		1.54	.462	9.35 × 10^‐3^	.491
Decanoylcarnitine	IVW	3	0.224	0.092	.0149	0.80	0.67–0.96	IVW and MR-Egger		1.74	.419	1.46 × 10^‐3^	.416
Citrate	IVW	6	0.189	0.037	<.0001	1.21	1.12–1.30	IVW and MR-Egger		2.73	.742	8.40 × 10^‐3^	.570

CI = confidence interval, IVW = inverse variance weighted, MR = Mendelian randomization, OR = odds ratio, Q = Cochran *Q* test statistics, SE = standard error, SNP = single-nucleotide polymorphism.

## 4. Discussion

To the best of our knowledge, the present research for the first time employed MR analysis on publicly available GWAS datasets to inform the causal effect of 7 serum metabolites, proline, serine, 3-dehydrocarnitine, N-[3-(2-oxopyrrolidin-1-yl)propyl]acetamide, octanoylcarnitine, decanoylcarnitine, and citrate, on cataract. Particularly, this research revealed that both serum 3-dehydrocarnitine and citrate have strong positive causal associations with cataract. Notably, 3-dehydrocarnitine is an intermediate metabolite involved in the biosynthesis and degradation of carnitine, which is crucial for the transport of medium- and long-chain fatty acids into mitochondria for beta-oxidation and energy production.^[23]^ While no direct evidence presents the involvement of 3-dehydrocarnitine in the development of cataract, we speculated that it may indirectly cause cataract through carnitine metabolism. Early studies have shown that carnitine has antioxidant properties via enhancing the antioxidant enzyme activities of catalase and glutathione peroxidase in a rat lens model,^[24]^ as well as suppressing lipid peroxidation and free radical scavenging in in vitro assays.^[25]^ As 3-dehydrocarnitine inversely correlates with carnitine, we postulated that 3-might be involved in cataract through dysregulations in carnitine biosynthesis and/or degradation and consequent oxidative stress. On the other hand, citrate is a well-known metabolite playing a key role in the citric acid cycle, which provides the majority of cellular energy through ATP production.^[26]^ However, current literature is controversial on the effect of citrate in the context of cataract. For instance, Nagai et al found that oral administration of citric acid to diabetic rats delayed the development of cataract,^[27]^ and Goulet et al found that citrate slowed the unfolding and aggregation of lens proteins.^[28]^ On the opposite, other studies showed that citrate-containing drugs led to retinal toxicities.^[29,30]^ Such discrepancy might be reconciled when cataract is caused by other chronic diseases with increased citrate levels, such as nonalcoholic fatty liver disease and type 2 diabetes.^[31,32]^ It is known that patients with nonalcoholic fatty liver disease or type 2 diabetes are at high risk of cataract,^[33,34]^ where the high blood level of citrate is a crucial biomarker for mitochondrial dysfunction and redox imbalance. In line with this, this research revealed that elevated serum citrate is a positive cataract risk factor.

Moreover, our analysis revealed that proline, serine, N-[3-(2-oxopyrrolidin-1-yl)propyl]acetamide, octanoylcarnitine, and decanoylcarnitine, have negative causal associations with cataract. Both proline and serine are nonessential amino acids. Proline is involved in protein synthesis and structure development, particularly in collagen formation.^[35]^ Early studies showed that proline effectively prevents the progression of cataract.^[36,37]^ It is postulated that the benign effect of proline stems from its involvement in collagen synthesis, a key substrate for maintaining the structural integrity of the lens, as well as its inhibitory effects on cellular apoptosis and oxidative stress.^[38,39]^ On the other hand, serine is integral to multiple metabolic pathways, such as the production of proteins, nucleotides, and lipids.^[40]^ Mounting evidence suggest that elevated serum serine has a protective role against cataract. Serine contributes to sphingolipid and phospholipid synthesis, which maintains lens cell membrane fluidity and lens transparency.^[41]^ Further, serine is a key precursor for glutathione synthesis, which is critical in the lens to prevent oxidative damage to crystallin proteins.^[42]^ Serine deficiency is therefore a recognized risk factor for cataract development, which agrees with this research. In addition, N-[3-(2-oxopyrrolidin-1-yl)propyl]acetamide, also known as acisoga, is an endogenous metabolite in human serum. It is still understudied in the context of cataract. Here, we found that N-[3-(2-oxopyrrolidin-1-yl)propyl]acetamide has a negative causal association with cataract, but the underlying mechanisms are still speculative and thus need further investigations. Notably, both octanoylcarnitine and decanoylcarnitine are medium-chain acylcarnitines, important in fatty acid metabolism and used as biomarkers for fatty acid metabolism efficiency. While the current literature lacks clinical and observational studies on octanoylcarnitine or decanoylcarnitine in cataract, early studies showed that elevated levels of octanoylcarnitine and decanoylcarnitine indicate altered mitochondrial function and antioxidant activities, which play critical roles in cataract pathogenesis. The direct or indirect mechanisms on the preventive roles of octanoylcarnitine and decanoylcarnitine on cataract require further investigations.

Like all other investigative studies, this research has limitations that must be considered. First, the GWAS data used were exclusively from individuals of European ancestry, which may hinder the generalization of the reported findings to other ethnic groups which present differential genetic backgrounds and baseline serum metabolite levels. Genetic variability may affect lens cell metabolisms and innate immune responses, potentially influencing the causal associations between serum metabolites and cataract. Future studies will be preferentially focused on incorporating multi-ethnic cohorts and stratified analyses based on genetic backgrounds are warranted to better elucidate the critical roles of serum metabolites in cataract pathogenesis and to validate the reported findings. Second, only 7 serum metabolites were studied in this research, however, approximately 4651 small molecule metabolites have been identified in human serum.^[43]^ As a result, changes in some serum metabolites can be secondary effects of upstream or more primary metabolic disturbances, which is a known issue in interpreting metabolomic data. Since this research has paved the road and demonstrated significance in the studied serum metabolites on cataract, future research might be focused on analyzing the causal effect of those serum metabolites that are central or closely relevant to the metabolic pathways involving the studied serum metabolites on cataract, for example, ornithine, arginine, acetylcarnitine, propionylcarnitine, palmitoylcarnitine, and stearoylcarnitine, which are closely relevant to the studied serum metabolites and involved in retinopathy. Last but not the least, MR analysis is a powerful tool for causal inference, but it does require strong assumptions and robust instrument selection. Thus, while our findings support significant causal associations between the studied serum metabolites and cataract, interpretations should remain cautious. Longitudinal studies and analyses of preclinical cataract cohorts are thereby needed to confirm whether alterations in serum metabolites precede disease onset.

**Figure 4.**
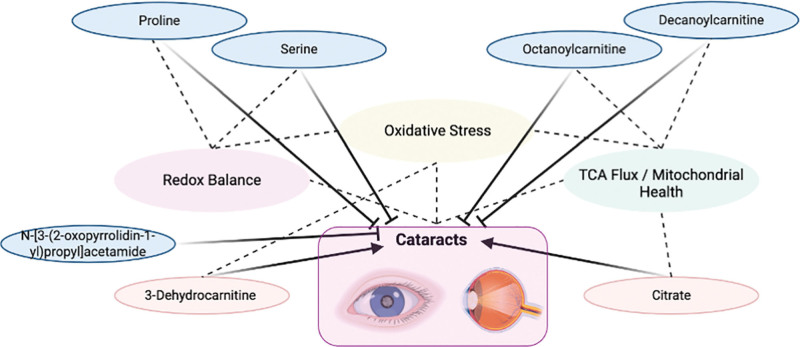
Schematic summary of pathway connections between the studied serum metabolites and cataract. Created with BioRender (https://www.biorender.com/).

In conclusion, this research employing MR analysis and provided evidence supporting significant causal of serum metabolites including proline, serine, 3-dehydrocarnitine, N-[3-(2-oxopyrrolidin-1-yl)propyl]acetamide, octanoylcarnitine, decanoylcarnitine, and citrate, on cataract (Fig. 4), which are largely in consistency with the current literature and advance the understanding of metabolism in cataract pathogenesis. The studied serum metabolites may serve as potential biomarkers for early disease diagnosis, but also represent potential therapeutic targets guiding future studies toward more effective management and treatment strategies for cataract.

## Acknowledgments

We acknowledge with gratitude the authors of the original datasets for making their data publicly available, which contributed to the analyses and conclusions of this study. Moreover, we sincerely acknowledge the time and effort from the anonymous reviewers, which have significantly contributed to the improved clarity and legibility of this work.

## Author contributions

**Conceptualization:** Xiaoying Yang, Jianzhong Mei.

**Formal analysis:** Xiaoying Yang, Jianzhong Mei.

**Investigation:** Xiaoying Yang, Jianzhong Mei.

**Methodology:** Xiaoying Yang.

**Software:** Xiaoying Yang.

**Supervision:** Jianzhong Mei.

**Validation:** Xiaoying Yang, Jianzhong Mei.

**Visualization:** Xiaoying Yang, Jianzhong Mei.

**Writing – original draft:** Xiaoying Yang.

**Writing – review & editing:** Jianzhong Mei.

## Supplementary Material


