# Supplementary material for: Investigating causal associations between serum metabolites and cataract by Mendelian randomization
Source: Medicine (Baltimore). 2026 Jan 2;105(1):e46662. doi: 10.1097/MD.0000000000046662 (PMC12778205; doi:10.1097/MD.0000000000046662)
Supplement: Supplementary file 1 [file medi-105-e46662-s001.docx]

**Supplemental Table 1. Serum metabolite datasets and instrumental variables.**

| **Dataset ID** | **Year** | **Exposure** | **Sample size** | **Number of SNPs** | **Selected SNPs** | **Range of F-statistics** | **Median F-statistic** |
| --- | --- | --- | --- | --- | --- | --- | --- |
| met-a-355 | 2014 | Proline | 7816 | 2545669 | rs11802885, rs2518802, rs5747934 | 29.63 - 261.78 | 196.5 |
| met-a-464 | 2014 | Serine | 7796 | 2545555 | rs1163251, rs4947534, rs715 | 58.12 - 122.26 | 86.31 |
| met-a-500 | 2014 | 3-Dehydrocarnitine | 7809 | 2545672 | rs2291429, rs273913, rs316019 | 31.19 - 108.96 | 41.74 |
| met-a-505 | 2014 | N-[3-(2-Oxopyrrolidin-1-yl)propyl]acetamide | 7812 | 2545675 | rs1005390, rs11101730, rs12602901, rs662138 | 52.93 - 117.12 | 77.18 |
| met-a-618 | 2014 | Decanoylcarnitine | 7766 | 2545676 | rs7552404, rs8396, rs924135 | 37.78 - 347.73 | 135.88 |
| met-a-615 | 2014 | Octanoylcarnitine | 7790 | 2545666 | rs2062541, rs7552404, rs8396 | 31.63 - 188.51 | 168.95 |
| met-c-849 | 2016 | Citrate | 24770 | 12086145 | rs1468269, rs172642, rs2040771, rs2921604, rs2954029, rs89356 | 30.73 - 107.53 | 54.91 |

**Supplemental Table 2. Additional MR analysis results employing MR-Egger, weighted median, simple mode and weighted mode methods.**

| **Exposure** | **Method** | **Number of SNP** | **Beta** | **SE** | **P-value** | **OR** |
| --- | --- | --- | --- | --- | --- | --- |
| Proline | MR Egger | 3 | 4.35 e-01 | 2.84 e-01 | 3.68 e-01 | 6.47 e-01 |
| Proline | Weighted median | 3 | 3.66 e-01 | 1.35 e-01 | 6.73 e-03 | 6.93 e-01 |
| Proline | Simple mode | 3 | 1.95 e-01 | 1.91 e-01 | 4.16 e-01 | 8.23 e-01 |
| Proline | Weighted mode | 3 | 3.36 e-01 | 1.68 e-01 | 1.84 e-01 | 7.14 e-01 |
| Serine | MR Egger | 3 | 7.32 e-01 | 2.34 e+00 | 8.07 e-01 | 4.81 e-01 |
| Serine | Weighted median | 3 | 5.50 e-01 | 2.36 e-01 | 1.96 e-02 | 5.77 e-01 |
| Serine | Simple mode | 3 | 5.55 e-01 | 2.81 e-01 | 1.87 e-01 | 5.74 e-01 |
| Serine | Weighted mode | 3 | 5.57 e-01 | 2.85 e-01 | 1.90 e-01 | 5.73 e-01 |
| 3-dehydrocarnitine | MR Egger | 3 | 3.41 e-01 | 1.64 e+00 | 8.70 e-01 | 1.41 e+00 |
| 3-dehydrocarnitine | Weighted median | 3 | 5.61 e-01 | 2.25 e-01 | 1.28 e-02 | 1.75 e+00 |
| 3-dehydrocarnitine | Simple mode | 3 | 7.08 e-01 | 3.16 e-01 | 1.54 e-01 | 2.03 e+00 |
| 3-dehydrocarnitine | Weighted mode | 3 | 6.41 e-01 | 2.51 e-01 | 1.25 e-01 | 1.90 e+00 |
| N-[3-(2-Oxopyrrolidin-1-yl)propyl]acetamide | MR Egger | 4 | 9.99 e-02 | 5.79 e-01 | 8.79 e-01 | 9.05 e-01 |
| N-[3-(2-Oxopyrrolidin-1-yl)propyl]acetamide | Weighted median | 4 | 2.62 e-01 | 1.53 e-01 | 8.75 e-02 | 7.70 e-01 |
| N-[3-(2-Oxopyrrolidin-1-yl)propyl]acetamide | Simple mode | 4 | 3.07 e-01 | 2.05 e-01 | 2.31 e-01 | 7.36 e-01 |
| N-[3-(2-Oxopyrrolidin-1-yl)propyl]acetamide | Weighted mode | 4 | 3.07 e-01 | 1.81 e-01 | 1.89 e-01 | 7.36 e-01 |
| Octanoylcarnitine | MR Egger | 3 | 3.87 e-01 | 1.91 e-01 | 2.92 e-01 | 6.79 e-01 |
| Octanoylcarnitine | Weighted median | 3 | 2.38 e-01 | 9.16 e-02 | 9.55 e-03 | 7.89 e-01 |
| Octanoylcarnitine | Simple mode | 3 | 2.49 e-01 | 1.12 e-01 | 1.57 e-01 | 7.80 e-01 |
| Octanoylcarnitine | Weighted mode | 3 | 2.40 e-01 | 9.89 e-02 | 1.36 e-01 | 7.86 e-01 |
| Decanoylcarnitine | MR Egger | 3 | 5.34 e-01 | 2.55 e-01 | 2.83 e-01 | 5.86 e-01 |
| Decanoylcarnitine | Weighted median | 3 | 2.60 e-01 | 1.00 e-01 | 9.64 e-03 | 7.71 e-01 |
| Decanoylcarnitine | Simple mode | 3 | 2.70 e-01 | 1.24 e-01 | 1.62 e-01 | 7.63 e-01 |
| Decanoylcarnitine | Weighted mode | 3 | 2.66 e-01 | 1.14 e-01 | 1.46 e-01 | 7.67 e-01 |
| Citrate | MR Egger | 6 | 8.77 e-02 | 1.68 e-01 | 6.30 e-01 | 1.09 e+00 |
| Citrate | Weighted median | 6 | 1.78 e-01 | 4.55 e-02 | 8.76 e-05 | 1.20 e+00 |
| Citrate | Simple mode | 6 | 1.81 e-01 | 6.18 e-02 | 3.29 e-02 | 1.20 e+00 |
| Citrate | Weighted mode | 6 | 1.69 e-01 | 5.34 e-02 | 2.51 e-02 | 1.18 e+00 |
